# Coir Fibers Treated with Henna as a Potential Reinforcing Filler in the Synthesis of Polyurethane Composites

**DOI:** 10.3390/ma14051128

**Published:** 2021-02-27

**Authors:** Sylwia Członka, Anna Strąkowska, Agnė Kairytė

**Affiliations:** 1 Faculty of Chemistry, Institute of Polymer and Dye Technology, Lodz University of Technology, −Stefanowskiego 12/16, 90-924 Lodz, Poland; anna.strakowska@p.lodz.pl; 2 Laboratory of Thermal Insulating Materials and Acoustics, Faculty of Civil Engineering, Institute of Building Materials, Vilnius Gediminas Technical University, Linkmenu St. 28, LT-08217 Vilnius, Lithuania; agne.kairyte@vilniustech.lt

**Keywords:** coir fibers, henna, polyurethane composites, UV-aging, mechanical performances

## Abstract

In this study, coir fibers were successfully modified with henna (derived from the *Lawsonia inermis* plant) using a high-energy ball-milling process. In the next step, such developed filler was used as a reinforcing filler in the production of rigid polyurethane (PUR) foams. The impact of 1, 2, and 5 wt % of coir-fiber filler on structural and physico-mechanical properties was evaluated. Among all modified series of PUR composites, the greatest improvement in physico-mechanical performances was observed for PUR composites reinforced with 1 wt % of the coir-fiber filler. For example, on the addition of 1 wt % of coir-fiber filler, the compression strength was improved by 23%, while the flexural strength increased by 9%. Similar dependence was observed in the case of dynamic-mechanical properties—on the addition of 1 wt % of the filler, the value of glass transition temperature increased from 149 °C to 178 °C, while the value of storage modulus increased by ~80%. It was found that PUR composites reinforced with coir-fiber filler were characterized by better mechanical performances after the UV-aging.

## 1. Introduction

Polyurethanes (PUR) represent a wide class of polymeric materials [1,2]. In recent years, the market for polymeric materials has shown strong, sustained growth and provided an opportunity for the integration of new bio-based feedstock for improved sustainability [3,4,5,6,7,8,9]. Agricultural waste has the potential to be utilized as renewable, bio-based fillers in the synthesis of PUR composites [10]. The use of agricultural waste as functionalized filler may not only improve the mechanical, physical, and combustion characteristics of PUR materials, but may also increase their biodegradability. Due to these positive and beneficial effects, it can be stated that the use of agricultural waste in the production of PUR materials will promote a new application path in converting agricultural waste into useful resources for creating a new class of green materials [11,12,13]. PUR materials derived from agricultural waste may offer solutions based on the main challenges of our times—the use of renewable raw materials, the economy and the preservation of resources, and minimization of the output of waste [14]. This approach creates an ideal scenario for the emergence of innovative opportunities under a rigid quality-control procedure that goes from the raw material to advanced polyurethane materials for construction and structural application.

In recent years, PUR composites reinforced with different natural fillers have been evaluated [15,16]. For example, PUR composite foams reinforced with flax and jute fibers were developed by Bledzki et al. [17]. The addition of flax fibers increased the mechanical performances of PUR composites. PUR composites reinforced with cellulose filler derived from eucalyptus pulp were synthesized by Silva et al. [18]. According to the scanning electron microscopy results, the incorporation of the filler up to 16 wt % resulted in a reduction in the size of composite cells and a deterioration of thermal conductivity. In another study, PUR composites were reinforced by selected amounts (5, 10, 15 wt %) of fly ash and wood ash, however, some deteriorations in mechanical performances were observed [19]. An opposite effect was observed by Olszewski et al. [20] in the case of PUR materials reinforced by the addition of glass and sisal fibers. In both cases, an improvement in the mechanical characteristics of PUR materials was observed. Cellulose derived from algal residue was selected as a reinforcing filler for PUR composites by Jonjaroen et al. [21]. According to the presented results, such developed PUR composites were characterized by greater apparent density, higher stiffness, and reduced loss modulus. Interesting results were reported by Li et al. in the case of PUR composites reinforced with bamboo fibers and alkali-treated bamboo fibers. The results showed that the alkali treatment of bamboo fibers resulted in better adhesion and interfacial interlocking between the fibers and PUR matrix, thus improving the mechanical performances of PUR composites [22].

Among different organic fillers, one of the most promising is coir fiber, which is extracted from coconut shells. Coir fibers possess many advantages, such as versatility and biodegradability [23]. Coir fibers constitute a byproduct during the processing of coconut, and the extraction of coir fibers is very easy and inexpensive. The obtained coir fibers have a high content of lignin (~50%) and low content of cellulose (~35%) [23]. Due to this, the coir fibers exhibit good mechanical properties, low density, and great thermal conductivity, making them an ideal candidate for application in different composites, such as concrete [24], natural rubber [25], unsaturated polyester resin [26], or epoxy composites [27]. According to the literature, 40 million metric tons of coconuts produces about 2 million metric tons of coir fibers; however, just a small fraction of coir-fiber waste is further reused as a reinforcing filler in composites [28]. Therefore, it seems logical and fully justified to use coir fibers in polymeric composites such as polyurethane foams, which are widely used in the building and construction sectors. However, the main concern connected with the application of cellulosic fillers is their low thermal stability [29]. Thus, the surface modification of the cellulosic filler seems to be necessary. Previous studies have reported different methods of filler modification, which include silane treatment [30], acetylation [31], or physical impregnation with selected flame-retardant compounds [32] or polysilsesquioxanes [33].

In our study, we propose a new kind of polyurethane (PUR) composites reinforced with coir fibers treated with henna, which is extracted from the *Lawsonia inermis* plant, commonly known as the henna tree. The main component of henna is lawsone (a hydroxynaphthoquinone), which possesses high antioxidative activity [34]. Besides this, henna contains various flavonoids, phenolic glycosides, quinoids, coumarins, xanthones, and tannins [35,36]. It is expected that due to the outstanding properties of coir fibers treated with henna, such reinforced PUR composites will be characterized by improved selected physico-mechanical properties. Therefore the impact of the selected content of coir fibers treated with henna on the mechanical, thermal, and antioxidative properties of PUR composites will be evaluated in the following study.

## 2. Materials and Methods

### 2.1. Materials

The materials used in the synthesis of PUR composites were as follows: Polymeric diphenylmethane diisocyanate (Purocyn B) as isocyanate (Purinova Company, Bydgoszcz, Poland); polyether polyol (Stapanpol PS-2352, Stepan Company, Northfield, IL, USA); potassium octoate (Kosmos 75) and potassium acetate (Kosmos 33) as catalysts (Evonik Industry, Essen, Germany); silicone-based surfactant (Tegostab B8513, Evonik Industry, Essen, Germany); pentane and cyclopentane as blowing agents (Sigma-Aldrich Corporation, Saint Louis, MO, USA); sodium hydroxide (Sigma-Aldrich Corporation, Saint Louis, MO, USA); nanopowder henna (Sigma-Aldrich Corporation, Saint Louis, MO, USA); and coir fibers (local company, Lodz, Poland).

### 2.2. Instruments and Methods

The dynamic viscosity of PUR systems was measured using a Viscometer DVII+ (Brookfield, Berlin, Germany) according to ISO 2555 [37]. The cellular structure of PUR composites was determined using scanning electron microscopy (SEM) (JEOL JSM 5500 LV, JEOL Ltd., Peabody, MA, USA). The content of closed cells was determined based on SEM images according to ISO 4590 [38]. The apparent density of PUR composites was calculated following ISO 845. The compressive test and flexural test were performed according to ISO 844 [39] and ISO 178 [40] using a Zwick Z100 testing machine (Zwick/Roell Group, Ulm, Germany). The thermogravimetric analysis (TGA) was performed using an STA 449 F1 Jupiter Analyzer (Netzsch Group, Selb, Germany) in the function of the temperature (0–600 °C). The UV-aging of PUR composites was performed using a UV 2000 (Atlas, Berwyn, PA, USA)—the samples were UV-irradiated for 7 days (radiation intensity = 0.7 W m^−2^, temperature = 60 °C). The color characteristic of PUR composites was determined using a CM-3600d spectrophotometer (Konica Minolta Sensing, Inc., Osaka, Japan).

### 2.3. Synthesis of PUR Composites

**Figure 1:**
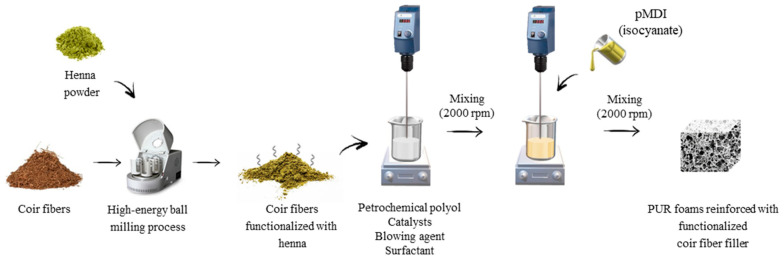
Schematic procedure of PUR composite preparation.

In the first step, coir fibers were alkali-treated following using NaOH solution (10% *v*/*v*). Subsequently, the alkali-treated coir fibers were functionalized with henna powder using a high-energy ball-milling process (60 min, 3000 RPM). The selected content of the developed coir-fiber filler (1, 2, and 5 wt %) was mixed with a polyol, water, surfactant, and catalysts using a mechanical stirrer (60 s, 2000 rpm). Subsequently, the polymeric diphenyl methane diisocyanate (pMDI) was added to the mixture and the PUR system was intensively mixed for another 30 s. The PUR composites were allowed to grow freely and left at room temperature for 48 h. The formulas of PUR composites are presented in Table 1. The schematic steps of PUR composites preparation are presented in Figure 1.

## 3. Results and Discussion

**Figure 2:**
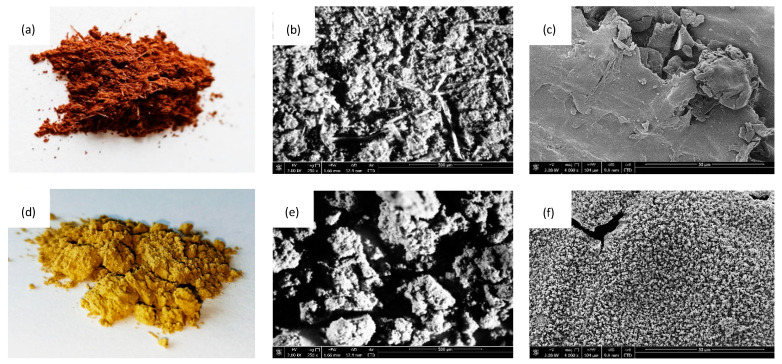
Optical and scanning electron microscope images of (**a**–**c**) pristine coir fibers and (**d**–**f**) coir-fiber filler treated with henna.

Scanning electron microscopy (SEM) of coir fibers before surface treatment and after the treatment with henna are presented in Figure 2. After the chemical treatment, the external surface of the coir-fiber filler seemed to be more rough than before the treatment. This may be connected with the alkali treatment, which removed the impurities and the waxes that smooth the filler surface. Moreover, it is believed that such a rough surface was connected with the presence of henna molecules, which created a coating layer on the filler surface. It is believed that such developed filler will be more compatible with PUR structure, providing better interfacial interlocking during PUR synthesis and avoiding the damages of PUR structure during the foaming process.

**Figure 3:**
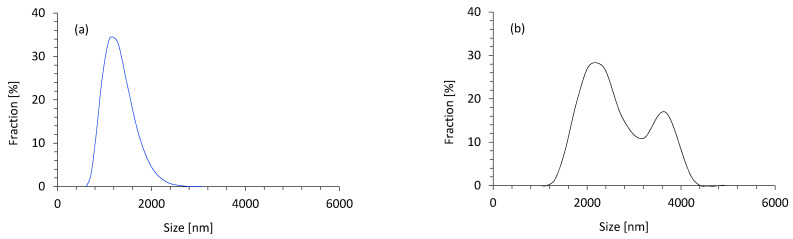
Particle-size distribution of (**a**) coir-fiber filler modified with henna and (**b**) pristine coir fibers.

The size of filler particles was determined using the dynamic light scattering (DLS) method. According to the results (Figure 3a), the coir-fiber filler treated with henna had 60.2% of particles with an average size between 1.0 and 1.5 μm. Apart from this, 8.5% of particles were in the range of 0.7–0.8 μm, and only 5.5% of particles were bigger than 1.5 µm. It can be seen that the application of a high-energy ball-milling process resulted in the formation of coir-fiber filler with a more homogenous particle-size distribution. Before the treatment, the pristine coir fibers were characterized by a less-uniform distribution of particle size, with different content of smaller and bigger particles. As shown in Figure 3b, most of the particles were in the 2.5–3.0 µm range; however, larger aggregates of coir-fiber filler with an average size of ~4.0 µm were also observed.

**Figure 4:**
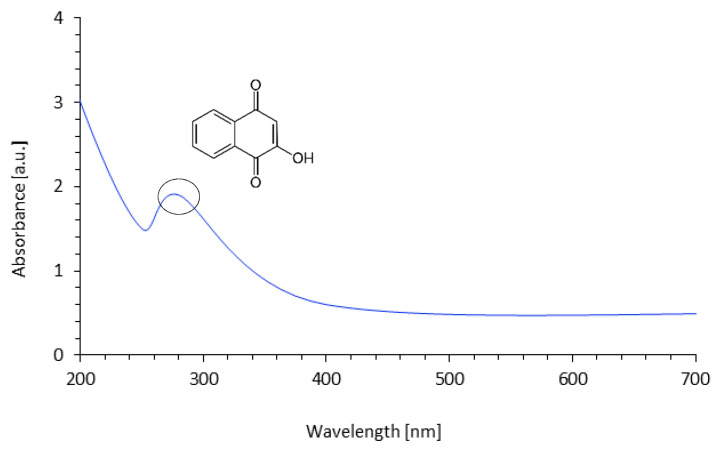
UV–VIS spectrum of coir-fiber filler treated with henna.

Ultraviolet–visible (UV–VIS) analysis of the coir-fiber filler treated with henna is presented in Figure 4. An intense peak at 280 nm corresponded to C=O and O-H groups of lawsone [35,41]. The long tail of the band beginning at 370 nm was associated with the yellow/orange color of the lawsone [41].

**Figure 5:**
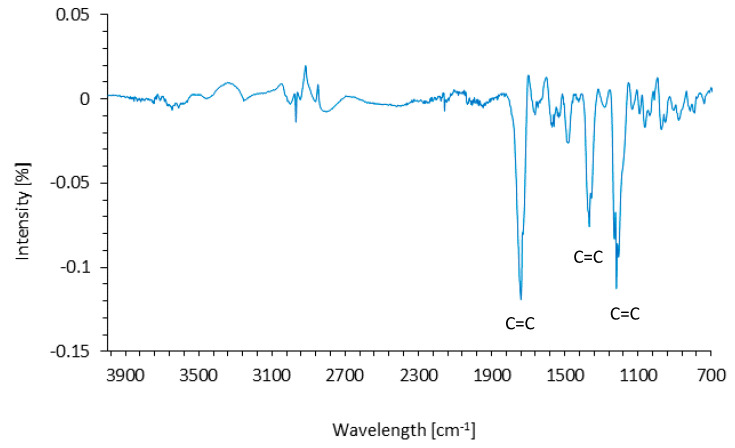
FTIR spectra of coir fibers treated with henna.

The FTIR spectrum of coir treated with henna is presented in Figure 5. The obtained spectrum showed the presence of three intense peaks at 1220, 1360, and 1740 cm^−1^, which correspond to the C=C aromatic of henna [42]. This indicates that the coir fibers were successfully functionalized with the henna compound, which effectively covered the surface of the fibers.

**Figure 6:**
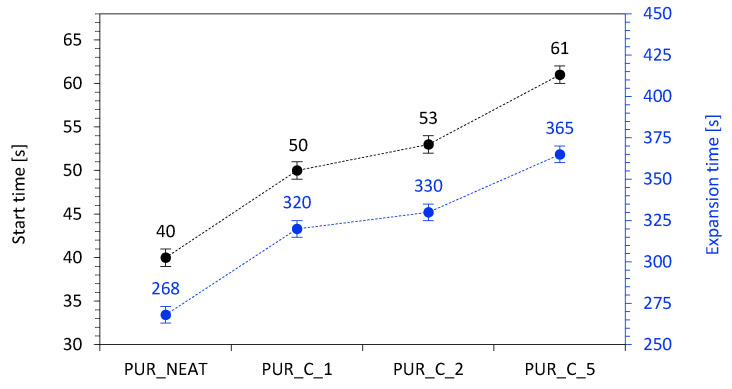
The results for processing times of the PUR systems.

The results of the foaming behavior of PUR composites were determined by measuring cream and expansion times (Figure 6). In this study, on the addition of coir-fiber filler, the cream time of PUR_C_1, PUR_C_2, and PUR_C_5 increased from 40 s to 50, 53, and 61 s, respectively, while the extension time increased from 268 s to 320, 330, and 365 s, respectively. These results can be explained based on the following aspects. PUR composites reinforced with coir-fiber filler were synthesized by chemical foaming with carbon dioxide (CO_2_) as the foaming agent, which is produced in the reaction between water and isocyanate groups. Since the addition of coir-fiber fillers could affect the proper stoichiometry of the reaction, the formation and releasing of CO_2_ was reduced, which resulted in limiting expansion of the cells and extended processing times [43,44]. Moreover, the further expansion of the cells was additionally hindered by the increased viscosity of PUR systems containing coir-fiber filler. Therefore, the addition of the filler resulted in extended processing times. Similar results can also be found in other studies [43,44].

**Figure 7:**
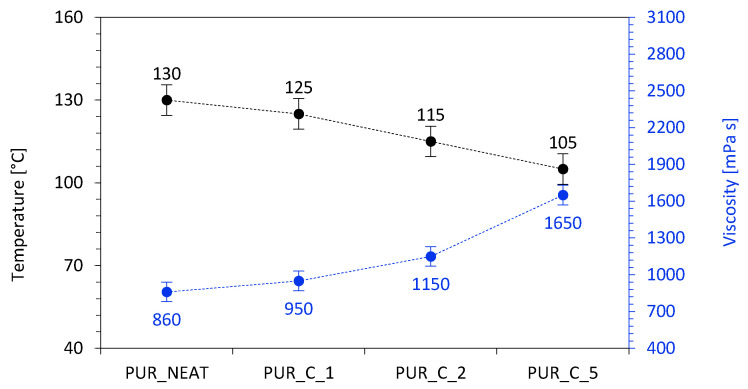
The results for temperature and dynamic viscosity of the PUR systems.

The addition of coir-fiber filler decreased the maximum temperature (T_max_) measured during the PUR synthesis (Figure 7). On the addition of 1, 2, and 5 wt % of the coir-fiber filler, the value of T_max_ decreased from 130 °C (for PUR_NEAT) to 125, 115, and 105 °C, respectively. This may be connected with the increased viscosity of the PUR systems with the addition of coir-fiber filler, which effectively reduced the efficiency of the reaction between functional groups of the components. Moreover, previous studies have shown that decreased temperature of PUR reinforced with natural fillers may be connected with the fact that during the foaming process, some heat is absorbed by the filler [45]. A similar explanation may be found in our study as well.

**Figure 8:**
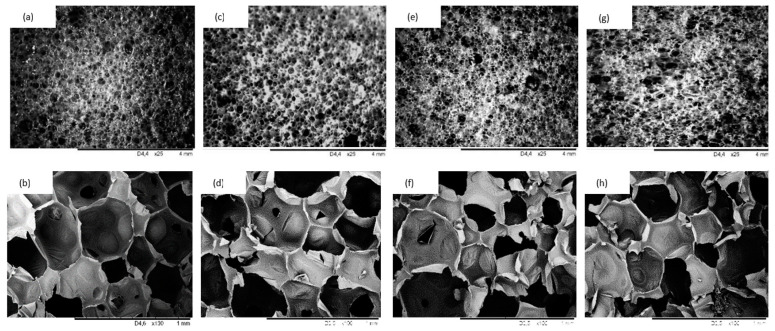
Scanning electron microscope images of (**a**,**b**) PUR_NEAT; (**c**,**d**) PUR_C_1; (**e**,**f**) PUR_C_2; (**g**,**h**) PUR_C_5.

The physico-mechanical properties of PUR composites are strongly affected by the rigidity of the PUR matrix and the cellular morphology of the composites. Previous studies have shown that, owing to the relatively large size of filler particles, the addition of fillers may disrupt the closed-cell structure of PUR composites, thereby altering their uniform integrity [46,47]. Therefore, the evaluation of the impact of coir-fiber filler on the cellular morphology of PUR composites seems to be necessary. As shown in Figure 8a, the overall structure of PUR_NEAT was quite uniform, with a high number of closed cells. On the addition of coir-fiber filler (Figure 8b–d), the closed-cell structure of PUR composites was still well preserved. Comparing all modified PUR composites, the highest number of broken cells and the most nonuniform size of the cells was exhibited by the PUR composite reinforced with 5 wt % of coir-fiber filler. When compared to PUR_NEAT, the percentage content of closed cells decreased from 85.2% to 78.6%. This can be attributed to poor compatibility between the hydrophilic filler and the hydrophobic PUR matrix, which resulted in collapse of the cells and the formation of open cells [48]. A more uniform structure was exhibited in the PUR composites reinforced with 1 and 2 wt % of coir-fiber filler. When compared with PUR_NEAT, the percentage content of closed cells increased slightly to 85.9 and 83.2% for PUR_C_1 and PUR_C_2, respectively. Moreover, with the addition of coir-fiber filler, the average size of the cells slightly decreased. The average size of the cells decreased from 490 μm (for PUR_NEAT) to 455, 440, and 430 μm for PUR_C_1, PUR_C_2, and PUR_C_5, respectively. This confirmed that the solid particles of the filler could act as a nucleating agent, changing the nucleation character from homo- to heterogeneous, and promoting the formation of a higher number of smaller cells [47,49,50].

**Figure 9:**
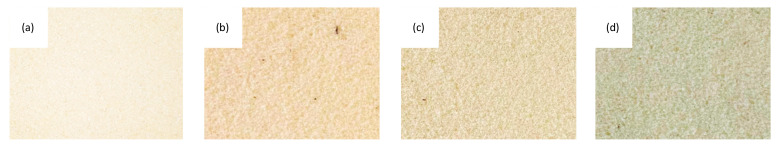
Optical images of (**a**) PUR_NEAT; (**b**) PUR_C_1; (**c**) PUR_C_2; (**d**) PUR_C_5

The impact of coir-fiber filler on the color of PUR composites was evaluated using the colorimetric analysis. According to the optical images presented in Figure 9, on the addition of the filler, the overall color of the PUR composites became greener. This was connected with the fact that henna contains a coloring matter—lawsone. According to the results of color analysis (Table 2), on the addition of the filler, the value of total color change became higher. Moreover, the values of a * and b *, which refer to the red/green and yellow/blue shades increased, indicating the more green and blue shadows of the PUR composites.

**Figure 10:**
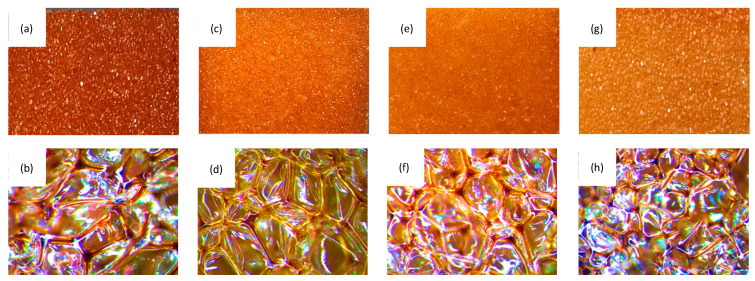
Optical images of (**a**,**b**) PUR_NEAT; (**c**,**d**) PUR_C_1; (**e**,**f**) PUR_C_2; (**g**,**h**) PUR_C_5.

Phytochemicals, especially phenols and flavonoids, are sensitive to UV-irradiation. The UV-irradiation can initiate a polymerization or degradation process of phytochemicals, leading to the deterioration of the physical and mechanical properties of the composites containing plant fillers. Therefore, the impact of controlled UV-irradiation on the structural and physical properties of PUR composites was evaluated. The optical images of PUR composites after the controlled UV-irradiation are presented in Figure 10. Based on this, it can be concluded that the addition of coir-fiber filler treated with henna had an impact on the color change of the PUR composites. The optical analysis revealed that after the UV-irradiation, the overall color of PUR composites had changed from yellow to orange, and this effect was most prominent in the case of PUR_NEAT. On the addition of coir-fiber filler, the difference in the color was not as intense. Confirmation of these results can be found in the color-parameter results (Table 2). When comparing all PUR composites, the most significant change in total color change (ΔE *) was observed for PUR_NEAT. On the addition of the coir-fiber filler, the difference in ΔE * was slightly reduced. A similar trend was observed in the case of the lightness parameter (L *)—on the addition of the coir-fiber filler, the value decreased. Moreover, the UV-aging increased the values of a * and b *, which refer to the red/green and yellow/blue shades. On the addition of coir-fiber filler, the differences between the parameters measured before and after the UV-aging were slightly reduced. This indicated that the addition of the coir-fiber filler treated with henna effectively protected the PUR composites from UV-radiation and acted as an antiaging compound. Good resistance of PUR composites against UV-irradiation should be attributed to the chemical composition of henna, which possesses strong antioxidant activity and successfully reduces a natural discoloration of PUR composites.

Mechanical properties are an important parameter that determines the further application of PUR composites. The compressive strength of PUR composites depends significantly on their apparent density. According to the results presented in Table 3, the incorporation of coir-fiber filler increased the apparent density of PUR composites, due to the incorporation of the filler with a certain weight. Depending on the content of coir-fiber filler, the value of apparent density increased from 35.9 kg m^−3^ to 37.9, 38.2, and 38.6 kg m^−3^ for PUR_C_1, PUR_C_2, and PUR_C_5, respectively. This may be explained by the following reasons. As discussed previously, PUR composites were synthesized in the foaming with CO_2_, which was formed in the reaction between water and isocyanate groups. Due to the filler incorporation, the viscosity of the PUR systems was increased, leading to a greater number of cells over a given volume. Therefore, the solid phases of PUR composites increased with the addition of the filler.

**Figure 11:**
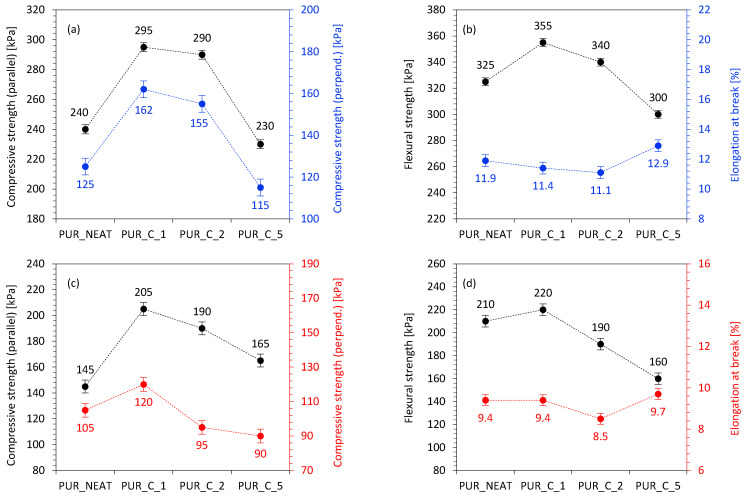
Mechanical performances of PUR composites measured (**a**,**b**) before, and (**c**,**d**) after the UV-aging.

The impact of the addition of coir-fiber filler on the σ_10%_ of the PUR composites is presented in Figure 11a. When compared to PUR_NEAT, the value of σ_10%_ increased by 23 and 21% for PUR_C_1 and PUR_C_2, respectively, and decreased by 4% for PUR_C_5. To eliminate the impact of apparent density on the value of σ_10%_, the compressive specific strength, defined as the ratio of σ_10%_ to the apparent density of the PUR composites, was evaluated as well. Depending on the content of coir-fiber filler, the specific compressive strength increased from 6.7 kPa/kg/m^3^ to 7.8 and 7.6 kPa/kg/m^3^ on the addition of 1 and 2 wt % of coir-fiber filler, respectively, and then decreased to 6.0 kPa/kg/m^3^ with a further increase of coir-fiber filler to 5 wt %. An analog dependency is observed in the case of flexural strength (σ_F_) (Figure 11b). When compared with PUR_NEAT, the value of σ_F_ increased by 9 and 5% for PUR_C_1 and PUR_C_2, respectively, and then decreased by 7% for PUR_C_5. Previous studies have reported that the mechanical characteristics of porous materials depend on interfacial interactions between filler surface and polymer matrix, as well as relatively uniform distribution of the filler in the composite structure [51]. A similar explanation can also be used in our study. The greatest improvement in mechanical characteristics was observed for PUR composites reinforced with 1 and 2 wt % of coir-fiber filler. Such a reinforcing effect may be connected with a uniform distribution of the coir-fiber filler in the PUR structure, as well as strong interphase adhesion between the functional groups of coir-fiber filler (mostly hydroxy groups of lawsone compound) and the PUR matrix. Therefore, the mechanical characteristic of the PUR composites was enhanced by a more rigid, cross-linked cellular structure. Furthermore, as discussed previously, the application of the high-energy ball-milling process resulted in the formation of smaller particles of the filler, which could be easily built in the PUR structure, showing a reinforcing function and effectively transferring an external load [52,53,54]. Owing to the higher stiffness of PUR composites, they were able to absorb more energy, effectively improving the mechanical resistance [55,56]. It has been shown that when the filler content was increased to 5 wt %, the mechanical properties were slightly decreased. According to previous studies, for a filler with an irregular shape, such as a cellulosic filler, the strength of the reinforced materials can be weakened by the filler’s insufficient ability to bear the stresses transferred from the matrix of the polymeric composites [55,56]. Besides, the high content of filler particles can result in poor interfacial adhesion, leading to the formation of partially separated microvoids between the polymer matrix and the filler surface, which prevent stress transfer. Due to the partial separation of the voids, the mechanical properties of PUR composites were deteriorated. According to the results presented in Figure 11c,d, the controlled UV-aging reduced the mechanical properties of the PUR composites. Interestingly, the most significant deterioration was observed in the case of PUR_NEAT. On the addition of coir-fiber filler, the mechanical properties were deteriorated as well; however, the difference was lower, as in the case of PUR_NEAT. The compressive strength decreased by ~36% for PUR_NEAT, while for the PUR composites reinforced with coir-fiber filler, the value decreased by ~28–34%, depending on the concentration of the filler. It seems that the solid particles of the filler may have acted as a physical barrier during the UV-aging and effectively supported the overall structure of the PUR composites, which resulted in better mechanical performances.

**Figure 12:**
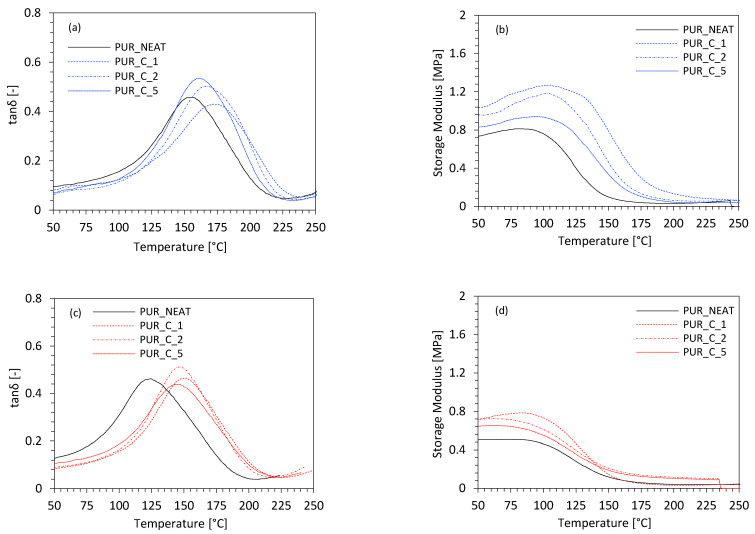
Dynamic-mechanical properties of PUR composites measured (**a**,**b**) before, and (**c**,**d**) after the UV-aging.

The dynamic mechanical properties (DMA), such as storage modulus (E′), and the glass-transition temperature (T_g_)—determined as a maximum peak on the graph of tanδ in the function of temperature—were evaluated. The results for storage modulus are presented in Figure 12a. When compared to the PUR_NEAT, on the incorporation of 1 and 2 wt % of coir-fiber filler, a significant improvement in E’ was observed. This may be explained by the following reasons: The alkali treatment removed the impurities and waxes on a fiber’s surface, and due to this, the fiber’s surface became more rough, increasing the interphase adhesion between the filler’s surface and the polymeric matrix. Furthermore, as discussed previously, on the incorporation of coir-fiber filler, the viscosity of the PUR systems was increased, limiting the mobility of the polymer chains, which resulted in the higher stiffness of the PUR structure. The incorporation of the coir-fiber filler increased the cross-linking density of PUR, due to the formation of chemical bridge bonds between functional groups of the coir-fiber filler and isocyanate groups. This induced the reinforcement effect and improved the overall mechanical characteristic of the PUR composites. On the addition of 5 wt % of coir-fiber filler, the dynamic mechanical properties of the PUR composites were slightly decreased. This indicates that the addition of coir-fiber filler in a higher concentration resulted in the formation of a more flexible structure in the PUR composites. The results for E’ were in agreement with the results for the glass-transition temperature (T_g_) (Figure 12b). When compared with PUR_NEAT, the value of T_g_ increased with the addition of the coir-fiber filler. Similar to the results for E’, the greatest improvement in T_g_ was observed for PUR_C_1—the value of T_g_ increased from 149 °C (for PUR_NEAT) to 178 °C. Such improvement in T_g_ observed for the PUR composites may be connected with the presence of the filler particles, which were built into the PUR structure and were able to create the interlocks between the PUR cells. Due to this, more energy was needed to reach the T_g_. The dynamic-mechanical properties of PUR composites subjected to the UV-aging were determined as well. According to the results presented in Figure 12c,d, after the UV-aging, all PUR composites were characterized by lower T_g_ and a reduced value of E’. Similar to the results for mechanical properties, the most significant decrease in dynamic-mechanical properties was observed for PUR_NEAT—the value of T_g_ decreased from 149 to 121 °C, while the E’ decreased by ~38%. On the addition of coir-fiber filler, the deterioration of the properties was not as significant. For example, in the case of PUR_C_5, the value of T_g_ decreased from 160 °C to 149 °C, while the value of E’ decreased by ~30%.

**Figure 13:**
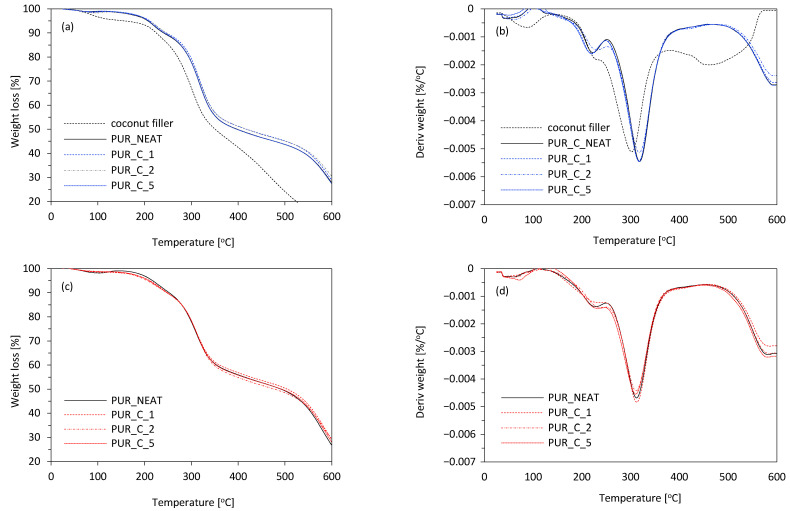
TGA results for PUR composites measured (**a**,**b**) before, and (**c**,**d**) after the UV-aging.

Thermal decomposition of the PUR composites was evaluated using thermogravimetric analysis (TGA) and derivative thermogravimetric analysis (DTGA). The results are shown in Table 4 and Figure 13.

In all cases, a three-stage decomposition of the PUR composites was observed. The first stage of decomposition (T_max1_) occurred in the range of 200–230 °C and referred to the moisture absorption of cellulosic filler [57,58,59,60] and decomposition of the urethane bond [57,58]. The second stage (T_max2_) of the PUR-composite decomposition occurred in the range of 300–350 °C, and should be assigned to the thermal pyrolysis of hard segments of PUR, which resulted in the formation of alcohol and isocyanate groups [61,62,63]. When compared with PUR_NEAT, T_max2_ increased on the addition of 1 and 2 wt % of coir-fiber filler—the value of T_max2_ increased from 312 °C (for PUR_NEAT) to 320 and 319 °C, respectively. Due to the more porous structure, the value of T_max2_ decreased slightly to 310 °C on the addition of 5 wt % of coir-fiber filler. The third stage of thermal decomposition of the PUR composites was mostly connected with the combustion of the composites, as well as thermal decomposition of the cellulosic derivatives hemicellulose and lignin [64,65]. When compared with PUR_NEAT, the PUR composites reinforced with 1 and 2 wt % of coir-fiber filler were more thermally stable—the value of T_max3_ increased from 585 °C (for PUR_NEAT) to 592 and 595 °C, respectively. The observed thermal-degradation behavior indicated that filler particles delayed the temperature of thermal decomposition by acting as a mass transport barrier and superior insulator to the volatile compounds, which were released during the thermal decomposition [66]. Besides, the thermal decomposition of PUR composites may be additionally reduced due to the higher cross-linking structure of the composites and the additional chemical bond between the functional groups of coir-fiber filler and isocyanate groups. The obtained results were in agreement with the results of the mass of char residue of the PUR composites obtained at 600 °C. Compared to PUR_NEAT, the mass of char residue increased from 29.2% to 30.3 and 30.9% for PUR_C_1 and PUR_C_2, respectively, and then decreased slightly to 28.7% for PUR_C_5. Based on this, it can be concluded that during the thermal-decomposition process, the filler particles could act as radical scavengers, while the higher cross-linking structure of the PUR composites effectively reduced heat transfer through the composite structure and improved their thermal resistance [66]. According to the results presented in Figure 13c,d, the UV-aging affected the thermal stability of the PUR composites; however, the differences between the maximum temperatures for non-aged and UV-aged PUR composites were not as significant.

## 4. Conclusions

In this study, coir fibers were successfully modified with henna (*Lawsonia inermis* plant) using a high-energy ball-milling process. In the next step, the developed filler was used as a reinforcing filler in the production of rigid polyurethane (PUR) foams. The impact of 1, 2, and 5 wt % of coir-fiber filler on the structural and physico-mechanical properties were evaluated. The obtained results revealed that depending on the filler content, an improvement or deterioration of the examined properties was observed. Among all modified series of PUR composites, the greatest improvement in physico-mechanical performances was observed for the PUR composite reinforced with 1 wt % of the coir-fiber filler. For example, on the addition of 1 wt % of coir-fiber filler, the compression strength was improved by 23%, while the flexural strength increased by 9%. Similar dependence was observed in the case of dynamic-mechanical properties—on the addition of 1 wt % of the filler, the glass-transition temperature increased from 149 °C to 178 °C, while the value of the storage modulus increased by ~80%. Interestingly, it was found that the PUR composites reinforced with coir-fiber filler were characterized by better mechanical performances after the UV-aging. Moreover, the incorporation of low thermally-stable cellulosic filler did not deteriorate the thermal stability of the PUR composites. When compared with the reference PUR composites, all series of modified PUR composites exhibited a similar degradation pattern. This led to the conclusion that the application of henna protected the coir-fiber filler and the reinforced PUR composites from UV-aging and high temperatures.

## Figures and Tables

**Table 1:** Formulas of PUR composites developed in the study.

Component	PUR_NEAT	PUR_C_1	PUR_C_2	PUR_C_5
	Parts by Weight (wt %) ^1^			
STEPANPOL PS-2352	100	100	100	100
PUROCYN B	160	160	160	160
Kosmos 75	6	6	6	6
Kosmos 33	0.8	0.8	0.8	0.8
Tegostab B8513	2.5	2.5	2.5	2.5
Water	0.5	0.5	0.5	0.5
Pentane/cyclopentane	11	11	11	11
Coir-fiber filler (coir fibers treated with henna)	0	1	2	5

^1^ Parts by weight (in relation to the weight of polyol (STEPANPOL PS-2352)).

**Table 2:** Color parameters of PUR composites measured before and after the UV-aging.

Sample	L *	a *	b *	ΔE *
	Before UV-Aging			
PUR_NEAT	14.3	63.4	−7.5	0
PUR_C_1	17.2	78.1	−3.7	14.6
PUR_C_2	22.5	78.3	−1.2	21.2
PUR_C_5	28.2	79.3	−0.2	25.2
	**After UV-Aging**			
PUR_NEAT	65.5	18.5	−4.1	50.2
PUR_C_1	55.4	31.1	−3.0	48.1
PUR_C_2	54.3	38.4	−2.9	42.5
PUR_C_5	51.1	49.6	0.5	41.5

ΔE *—total color change, L *—white/dark degree, a *—red/green shade, b *—yellow/blue shade.

**Table 3:** Cell size, closed-cell content, and apparent density of PUR composites before and after UV-aging.

Sample	Before UV-Aging			After UV-Aging			
	Cell Size [µm]	Closed-Cell Content [%]	Apparent Density [kg m^−3^]		Cell Size [µm]	Closed-Cell Content [%]	Apparent Density [kg m^−3^]
PUR_NEAT	490	85.2	35.9		505	75.4	31.3
PUR_C_1	455	85.9	37.9		470	76.2	33.5
PUR_C_2	440	83.2	38.2		455	75.2	33.7
PUR_C_5	430	78.6	38.6		450	70.2	35.8

**Table 4:** The results of the thermogravimetric and derivative thermogravimetric analyses.

Sample Codes	T_10%_ [°C]	T_50%_ [°C]	T_80%_ [°C]	Char Residue (at 600 °C) [%]
	Before UV-Aging			
PUR_NEAT	213	312	585	29.2
PUR_C_1	215	320	592	30.3
PUR_C_2	225	319	595	30.9
PUR_C_5	210	310	576	28.7
	**After UV-Aging**			
PUR_NEAT	227	309	579	28.3
PUR_C_1	230	313	582	28.9
PUR_C_2	231	315	585	28.8
PUR_C_5	231	308	586	28.2

## Data Availability

Data sharing not applicable.
